# A novel C-terminal heat shock protein 90 inhibitor that overcomes STAT3-Wnt-β-catenin signaling-mediated drug resistance and adverse effects

**DOI:** 10.7150/thno.63788

**Published:** 2022-01-01

**Authors:** Ho Jin Lee, Hye-Young Min, Young-Sik Yong, Jihyae Ann, Cong Truong Nguyen, Minh Thanh La, Seung Yeob Hyun, Huong Thuy Le, Hyewon Kim, Hyukjin Kwon, Gibeom Nam, Hyun-Ju Park, Jeewoo Lee, Ho-Young Lee

**Affiliations:** 1 Creative Research Initiative Center for concurrent control of emphysema and lung cancer, College of Pharmacy, Seoul National University, Seoul 08826, Republic of Korea.; 2 College of Pharmacy and Research Institute of Pharmaceutical Sciences, Seoul National University, Seoul 08826, Republic of Korea.; 3 Department of Molecular Medicine and Biopharmaceutical Sciences, Graduate School of Convergence Science and Technology and College of Pharmacy, Seoul National University, Seoul 08826, Republic of Korea.; 4 School of Pharmacy, Sungkyunkwan University, Suwon, Gyeonggi-do 16419, Republic of Korea.

**Keywords:** heat shock protein 90, antitumor, deguelin, drug resistance

## Abstract

**Rationale:** The heat shock protein (Hsp) system plays important roles in cancer stem cell (CSC) and non-CSC populations. However, limited efficacy due to drug resistance and toxicity are obstacles to clinical use of Hsp90 inhibitors, suggesting the necessity to develop novel Hsp90 inhibitors overcoming these limitations.

**Methods:** The underlying mechanism of resistance to Hsp90 inhibitors was investigated by colony formation assay, sphere formation assay, western blot analysis, and real-time PCR. To develop anticancer Hsp90 inhibitors that overcome the signal transducer and activator of transcription 3 (STAT3)-mediated resistance, we synthesized and screened a series of synthetic deguelin-based compounds in terms of inhibition of colony formation, migration, and viability of non-small cell lung cancer (NSCLC) cells and toxicity to normal cells. Regulation of Hsp90 by the selected compound NCT-80 [5-methoxy-N-(3-methoxy-4-(2-(pyridin-3-yl)ethoxy)phenyl)-2,2-dimethyl-2H-chromene-6-carboxamide] was investigated by immunoprecipitation, drug affinity responsive target stability assay, binding experiments using ATP-agarose beads and biotinylated drug, and docking analysis. The antitumor, antimetastatic, and anti-CSC effects of NCT-80 were examined *in vitro* and *in vivo* using various assays such as MTT, colony formation, and migration assays and flow cytometric analysis and tumor xenograft models.

**Results:** We demonstrated a distinct mechanism in which Hsp90 inhibitors that block N-terminal ATP-binding pocket causes transcriptional upregulation of Wnt ligands through Akt- and ERK-mediated activation of STAT3, resulting in NSCLC cell survival in an autocrine or paracrine manner. In addition, NCT-80 effectively reduced viability, colony formation, migration, and CSC-like phenotypes of NSCLC cells and their sublines with acquired resistance to anticancer drugs by inducing apoptosis and inhibiting epithelial-mesenchymal transition and the growth of NSCLC patient-derived xenograft tumors without overt toxicity. With regards to mechanism, NCT-80 directly bound to the C-terminal ATP-binding pocket of Hsp90, disrupting the interaction between Hsp90 and STAT3 and degrading STAT3 protein. Moreover, NCT-80 inhibited chemotherapy- and EGFR TKI-induced programmed cell death ligand 1 expression and potentiated the antitumor effect of chemotherapy in the LLC-Luc allograft model.

**Conclusions:** These data indicate the potential of STAT3/Wnt signaling pathway as a target to overcome resistance to Hsp90 inhibitors and NCT-80 as a novel Hsp90 inhibitor that targets both CSCs and non-CSCs in NSCLC.

## Introduction

Non-small cell lung cancer (NSCLC), which accounts for 80%-85% of all lung cancers, is one of the major causes of cancer-related deaths worldwide 1. Despite recent advances in therapeutics, such as molecular targeted therapies and immunotherapies, and the integration of personalized medicine 2, the benefits gained from these newly developed therapies is minimal for most patients due to primary drug resistance 3. Moreover, the occurrence of acquired drug resistance is an inevitable consequence in most primarily susceptible patients 3. Thus, the development of anticancer drugs that block multiple molecular targets involved in several oncogenic signaling pathways rather than a single target in one specific signaling pathway would be an effective therapeutic strategy.

The heat shock protein (Hsp) system plays a central role in cellular homeostasis under various extracellular and intracellular insults 4, 5. The Hsp system also controls the conformational maturation and stability of various oncoproteins associated with the hallmarks of cancer 5, 6. Recent studies have revealed the role of Hsp system in maintaining the functional features of cancer stem cells (CSCs), a rare subpopulation within the tumor defined by their capacity for self-renewal and generation of primary, recurrent, and metastatic tumors with heterogeneity and anticancer drug resistance 4, 7. Among a number of components, Hsp90 is frequently overexpressed in NSCLC and contributes to poor prognosis of these patients 8. Hence, Hsp90-targeting anticancer drugs could be effective for eliminating both CSCs and non-CSC populations in NSCLC, thereby treating NSCLC. Indeed, various Hsp90 inhibitors with different structural backbones, especially those that block N-terminal ATP-binding pocket, have been developed 9, 10. These inhibitors have shown potent anticancer activities in preclinical settings 10; however, none of them have been approved for clinical use due to limited therapeutic efficacy 11, 12, ocular and liver toxicities 12, and/or emergence of drug resistance.

It has been suggested that dying cells after chemotherapy and/or radiation therapy release soluble factors that promote the survival of neighboring live cells 13. Wnt/β-catenin signaling is one of the essential pathways for stem-like properties, including self-renewal and differentiation 14. Studies have shown that the aberrant activation of Wnt/β-catenin signaling plays an important role in CSCs and anticancer drug resistance 15, 16, and therapy-induced WNT16B secretion promotes cell proliferation and chemoresistance 17. Autocrine or treatment-induced feedback activation of signal transducer and activator of transcription 3 (STAT3) also mediates cell survival and drug resistance 18, 19. STAT3-mediated transcriptional network was found to stimulate CSC function and chemoresistance in multiple types of cancers, including NSCLC 20. Previous studies have shown a collaborative interaction between the Wnt- and STAT3-mediated signaling pathways for tumor initiation and metastasis 18, 20-23. However, role of the functional crosstalk between Wnt- and STAT3-mediated signaling in the emergence of drug resistance remains elusive.

In the present study, we aimed to understand the underlying mechanism of resistance to Hsp90-targeting anticancer drugs and develop novel Hsp90 inhibitors for anticancer therapy. We show that compensatory activation of STAT3 through the protein kinase B/extracellular signal-regulated kinase (Akt/ERK)-mediated transcriptional upregulation of interleukin 6 (IL6) stimulates Wnt/β-catenin signaling, causing resistance to Hsp90-targeting anticancer drugs, especially those targeting the N-terminal ATP-binding pocket. We synthesized 5-methoxy-N-(3-methoxy-4-(2-(pyridin-3-yl)ethoxy)phenyl-2,2-dimethyl-2H-chromene-6-carboxamide, designated as NCT-80, as a safe and efficient Hsp90 inhibitor. NCT-80 has broad and potent antitumor effects on both CSCs and non-CSCs in NSCLC *in vitro* and *in vivo* without overt toxicity and compensatory activation of the STAT3 and Wnt/β-catenin pathway. Furthermore, NCT-80 inhibited programmed cell death ligand 1 (PD-L1) expression in chemoresistant cells and enhanced antitumor effects of chemotherapeutic agents. Our results demonstrate that NCT-80 is a highly potent anticancer drug targeting C-terminal ATP-binding pocket of Hsp90.

## Methods

### Reagents

Antibodies specific for c-Myc, Bcl-2, cyclin D1, survivin, Bax, Twist, and actin were purchased from Santa Cruz Biotechnology (Dallas, TX, USA). Antibodies against phosphorylated LRP6 (pLRP6, S1490), LRP6, Dvl3, β-catenin, Oct4, Nanog, Sox2, Histone H3, Snail, Slug, phosphorylated Akt (pAkt, S473), Akt, phosphorylated ERK (pERK, T202/Y204), ERK, STAT3, phosphorylated STAT3 (pSTAT3, Y705), Tubulin, GAPDH, PD-L1, IL6, and cleaved caspase-3 (Cl-Cas3) were purchased from Cell Signaling Technology (Danvers, MA, USA). Antibodies against cleaved PARP, E-cadherin, and N-cadherin were purchased from BD Biosciences (San Jose, CA, USA). Horseradish peroxidase (HRP)-conjugated anti-rabbit, anti-mouse, and anti-goat secondary antibodies were purchased from GeneTex (Irvine, CA, USA). An anti-proliferating cell nuclear antigen (PCNA) antibody was purchased from Abcam (Cambridge, UK). Anti-Hsp90 and anti-Hsp70 antibodies were purchased from Enzo Life Science (Farmingdale, NY, USA). Ni-NTA agarose was purchased from Invitrogen (Carlsbad, CA, USA) and ATP-agarose was acquired from Innova Biosciences (Cambridge, UK). A first-strand cDNA synthesis kit was purchased from Transgen Biotech (Beijing, China). M50 Super 8x TOPFlash (Addgene plasmid #12456; http://n2t.net/addgene:12456; RRID:Addgene_12456) and M51 Super 8x FOPFlash (TOPFlash mutant) (Addgene plasmid #12457; http://n2t.net/addgene:12457; RRID:Addgene_12457) were gifts from Randall Moon 24. An expression vector for the constitutively activated form of STAT3 (CA-STAT3) was kindly provided by Dr. Sang-Kyu Ye (Seoul National University, Seoul, Republic of Korea). Propidium iodide (PI), 3-(4,5-dimethylthiazol-2-yl)-2,5-diphenyltetrazolium bromide (MTT), crystal violet, avidin-agarose, an anti-vimentin antibody, and other chemicals were purchased from Sigma-Aldrich (St. Louis, MO, USA) unless otherwise specified.

### Cell culture

Human NSCLC cell lines (H1299, A549, H460, H226B, and PC9) were purchased from the American Type Culture Collection (ATCC, Manassas, VA, USA) or kindly provided by Dr. John V. Heymach (The University of Texas M. D. Anderson Cancer Center, Houston, TX, USA). Human retinal pigment epithelial (RPE) cells were kindly provided by Dr. Jeong Hun Kim (College of Medicine, Seoul National University, Seoul, Republic of Korea). HT-22 mouse hippocampal neuronal cells were kindly provided by Dr. Dong Gyu Jo (College of Pharmacy, Sungkyunkwan University, Suwon, Republic of Korea). L-Wnt3a cells were kindly provided by Dr. Sang Kook Lee (Seoul National University). Drug resistant H1299/CsR, H1299/PmR, H460/PcR, and PC9/ER cells were generated by continuous exposure to increasing concentrations of cisplatin (H1299/CsR), pemetrexed (H1299/PmR), paclitaxel (H460/R), or erlotinib (PC9/ER) for more than six months 25-27. The NSCLC cell lines were authenticated and validated in 2013 at the Korean Cell Line Bank using an AmplFLSTR Identifier PCR Amplification Kit (Applied Biosystems, Foster, CA, USA; cat. No. 4322288). NSCLC cells were cultured in RPMI 1640 medium supplemented with 10% fetal bovine serum (FBS) and antibiotics, all purchased from Welgene Inc. (Gyeongssan-si, Republic of Korea). RPE and HT-22 cells were cultured in Dulbecco's Modified Eagle Medium (DMEM; Welgene) supplemented with 10% FBS and antibiotics. Cells were incubated at 37 °C with 5% CO_2_ in a humidified atmosphere. Cells passed less than 6 months after receipt or recovery of validated cells without mycoplasma contamination were used in the study.

### MTT assay

NSCLC cells, RPE cells, or HT-22 cells (2-3 × 10^3^ cells/well in 96-well plates) were treated with increasing concentrations of NCT-80 (0.1, 1, and 10 μM) for 72 h. MTT solution was then added to the cells at a final concentration of 200 μg/mL and incubated for 2 h at 37 °C. The formazan products were dissolved in dimethyl sulfoxide (DMSO) and the absorbance measured at 570 nm. The data are presented as a percentage of that of the control group.

### Anchorage-dependent and anchorage-independent colony formation

Anchorage-dependent and anchorage-independent colony formation assays were performed as described previously 25. To determine the effect of NCT-80 on anchorage-dependent colony formation, NSCLC cells were plated at a density of 300 cells/well in six-well plates and treated with various concentrations of NCT-80 for 1.5-2 weeks. The developed colonies were fixed with 100% methanol, stained with 0.02% crystal violet solution, photographed, and counted.

To determine the effect of NCT-80 on anchorage-independent colony formation, cells were mixed with sterile 1% agar solution (final concentration of 0.4%) and then poured onto 1% base agar in 12- or 24-well plates. After solidification, test compounds diluted in complete medium were added to the agar overlays and incubated for 2 weeks. Colonies were then stained with MTT solution, photographed, and counted using ImageJ software (National Institutes of Health, Bethesda, MA, USA).

### Sphere formation assay

NSCLC cells were seeded on ultra-low attachment 96-well plates (Corning, Corning, NY, USA) in spheroid medium [DMEM-F12, supplemented with B27 supplements (Thermo Fisher Scientific, Waltham, MA, USA), EGF, bFGF, and antibiotics]. After seeding, cells grown under the aformentioned sphere-forming conditions were treated with vehicle or NCT-80 (up to 1 μM) and were incubated at 37 °C and 5% CO_2_ for 2 weeks or until spheres formed and reached sizes above 150 µm^3^.

### Wound-healing assay

H1299 or A549 cells were seeded into 6-well plates (2 × 10^5^ cells/well). Once the cells reached confluence, the cell monolayer was scratched using a sterile yellow micro pipette tip. After being photographed, the cells were treated with vehicle or test compounds for up to 24 h and then photographed again. The extent of wound healing was determined by analyzing the photographs using ImageJ software.

### Cell cycle analysis

NSCLC cells were treated with vehicle (DMSO) or 5 μM NCT-80 for two days. All adherent or floating cells were collected and washed with phosphate-buffered saline (PBS). The cells were then fixed with 100% methanol and stained with 50 μg/mL PI solution containing 50 μg/mL RNase A for 30 min at room temperature. Changes in cell cycle distribution were determined by flow cytometry using a FACSCalibur^®^ flow cytometer (BD Biosciences).

### Reporter gene assay

To determine the effect of NCT-80 on β-catenin/Tcf transcriptional activity, a reporter gene assay was performed using a Beetle Juice Luciferase Assay Kit (PJK GmbH, Kleinblittersdorf, Germany) according to the manufacturer's protocol. Briefly, cells were co-transfected with vectors M50 Super 8x TOPFlash or M51 Super 8x FOPFlash and pSV-β-Galactosidase. After treatment with the test compounds in the presence or absence of Wnt3a conditioned media (CM) obtained from L-Wnt3a cell cultures, cells were harvested with lysis buffer and luciferase activity monitored using an M5 SpectraMax Microplate Reader (Molecular Devices, Sunnyvale, CA, USA). Transfection efficiency was normalized by measuring β-galactosidase activity using β-Gal Juice (PJK GmbH).

### Transfection

For transient transfections, cells were transfected with the indicated expression vectors or small interfering RNAs (siRNAs) targeting *IL6* (purchased from Bioneer Corp., Daejeon, Republic of Korea) using jetPrime transfection reagent (Polyplus-Transfection SA, Illkirch, France) according to the manufacturer's recommended procedure. For siRNA transfection, cells were seeded in 60-mm culture dish, and transfected with 60 pmoles of the scrambled or *IL6* siRNAs. After 24 h, the medium was changed with fresh complete medium.

### Western blot analysis

Cells were harvested with RIPA lysis buffer as described previously 28. For preparing cytoplasmic and nuclear extracts, cells were scrapped and washed with ice-cold PBS. Cells were pelleted by centrifugation at 5,000 rpm for 7 min at 4 °C. Pellets were resuspended in 100-200 μL of buffer A [10 mM HEPES (pH 7.9), 10 mM KCl, 1.5 mM MgCl_2_, 1 mM DTT, 1 mM PMSF, 0.1% NP-40, 1 μg/mL leupeptin, and 1 μg/mL aprotinin] and incubated on ice for 10 min. The samples were centrifuged at 3,000 rpm for 10 min at 4 °C. The supernatants were collected and used as cytoplasmic extracts. After washing twice with buffer A, pellets were further resuspended with 60 μL of buffer C [20 mM HEPES (pH 7.9), 400 mM NaCl, 0.2 mM EDTA, and 25% Glycerol]. The samples were incubated on ice over 15 min with frequent vortexing. The homogenates were centrifuged at 13,200 rpm for 20 min at 4 °C. The supernatants were collected and used as nuclear extracts. Equal amounts of cell lysates and cytoplasmic/nuclear extracts were resolved by 8-15% sodium dodecyl sulphate-polyacrylamide gel electrophoresis (SDS-PAGE) and transferred onto a polyvinylidene difluoride (PVDF) membrane. Membranes were blocked for 1 h at room temperature with blocking buffer consisting of 3% skim milk in tris-buffered saline containing 0.1% Tween-20 (TBST). The blocked membranes were then incubated with primary antibodies diluted in TBST containing 3% bovine serum albumin (BSA) (a 1:1,000 dilution) overnight at 4 °C. Membranes were washed three times with TBST for 1 h at room temperature and then incubated with the corresponding secondary antibodies diluted in 3% skim milk in TBST (a 1:5,000 dilution) for 1 h at room temperature. Membranes were washed three times with TBST and visualized using an enhanced chemiluminescence (ECL) detection kit (Thermo Fisher Scientific).

### Preparation of recombinant proteins, immunoprecipitation, and pull-down assay

Cloning and purification of recombinant Hsp90 and Hsp70 proteins and immunoprecipitation and pulldown assays to determine the interaction between NCT-80 and Hsp90 were performed as described in our previous reports 28, 29

### Drug affinity responsive target stability (DARTS)

DARTS was performed according to the previously published procedure with some modifications 29. Briefly, 35 μg of purified Hsp90 and Hsp70 proteins were treated with vehicle (DMSO) or 5 μM NCT-80 (final 1% DMSO) for 30 min at 4 °C, and then incubated with proteinase K (proteinase K:protein ratio 1:10 and 1:40) for 15 min at room temperature. After adding 5x SDS-PAGE sample buffer and boiling for 5 min at 95 °C, lysates were separated by 8% SDS-PAGE, transferred onto PVDF membranes, and further subjected to Western blot analysis as described above.

### Real-time polymerase chain reaction (PCR)

Cells were treated with various concentrations of NCT-80 for 24 h. Total RNA was prepared using an easy-BLUE total RNA extraction kit (Intron Biotechnology, Sungnam-si, Kyunggi-do, Republic of Korea) according to the manufacturer's protocol. SYBR Green-based real-time PCR analysis was performed according to the protocol provided by the manufacturer (Enzynomic, Daejeon, Republic of Korea). The primer sequences used for real-time PCR are listed in **Table S1**. The thermocycler conditions for real-time PCR were as follows: pre-incubation at 95 °C for 10 min, 40-50 cycles of 95 °C for 10 s, 60 °C for 10 s, and 72 °C for 10 s, and melting curve analysis was performed to determine reaction specificity. Relative quantification of mRNA expression was performed by the comparative CT (cycle threshold) method.

### Immunofluorescence staining

Cells were seeded onto coverslip and then treated with test materials for 24 h. Cells were fixed with 4% paraformaldehyde for 10 min at room temperature, washed with PBS, and then permeabilized with 0.3% Triton X-100 for 15 min at room temperature. Cells were washed with PBS and then incubated with blocking solution [3% bovine serum albumin (BSA) in Tris-buffered saline containing 0.1% Tween-20] for 1 h at room temperature. Cells were incubated with primary antibodies (a 1:200 dilution) at 4ºC overnight. Cells were washed several times with PBS and incubated with Alexa Fluor 594-conjugated secondary antibodies (Thermo Fisher Scientific, a 1:1,000 dilution) for 1 h at room temperature. Cells were washed multiple times with PBS and counterstained with 4′,6-diamidino-2-phenylindole (DAPI). The coverslips were mounted with mounting solution (Dako, Glostrup, Denmark) and then observed under a fluorescence microscope (Zeiss Axio Observer Z1, Carl Zeiss AG, Oberkochen, Germany).

### Animal experiments

All animal experiments were conducted using protocols approved by the Seoul National University Institutional Animal Care and Use Committee. Mice were freely fed standard mouse chow and water and housed in a temperature- and humidity-controlled facility with a 12-h light/12-h dark cycle. For the xenograft experiments, NSCLC patient-derived tumors (5 x 10^6^ cells/spot) 26 were subcutaneously injected into the right flank of 6-week-old male and female NOD/SCID mice. For the allograft experiments, LLC-Luc cells were subcutaneously injected into the right flank of 8-week-old female C57BL/6 mice. After the tumor volume had reached 50-150 mm^3^, the mice were randomly assigned to different experimental groups (*n* = 6 per group). Six times per week for 3 weeks, the animals were orally administered vehicle (10% DMSO in corn oil) or NCT-80 (50 mg/kg). For drug combination, mice were administered either NCT-80 (oral gavage) or anticancer therapeutics (Pc/Cs [a combination of paclitaxel (20 mg/kg, dissolved by adding solvents to the paclitaxel powder individually and in order: 10% DMSO, 40% PEG300, 5% Tween 80, and distilled water) and cisplatin (3 mg/kg, dissolved in 0.9% NaCl solution)], once a week by intraperitoneal administration). Tumor growth was determined by measuring the short and long diameter of the tumor using a caliper, and body weight was measured twice per week to monitor toxicity. The tumor volume was calculated using the following formula: tumor volume (mm^3^) = (small diameter)^2^ × (large diameter) × 0.5.

### Serial dilution tumor-propagating assay

NOD/SCID mice bearing H460 xenograft tumors were treated with vehicle or NCT-80 (50 mg/kg) for 16 days. Tumors were dissociated, and the isolated primary tumor cells were serially diluted and subcutaneously inoculated into the right flank of 6-week-old male and female NOD/SCID mice (5 × 10^2^ - 5 × 10^4^ viable cells per spot). The incidence of tumor formation was monitored using physical palpation. Tumor-initiation fraction of vehicle- or NCT-80-treated cells was analyzed by using Extreme Limiting Dilution Analysis (ELDA) online software (http://bioinf.wehi.edu.au/software/elda/).

### Toxicity test

For the toxicity tests, FVB mice were orally administered vehicle or NCT-80 (50 mg/kg) every day for 2 weeks. Blood samples from mice were collected by retro-orbital puncture (ROP) technique. After allowing blood coagulation at 4 °C, serum was collected by centrifugation at 3000 rpm for 10 min at 4 °C. Analysis of serum levels of blood urea nitrogen (BUN), creatinine, glutamate oxaloacetate transaminase (GOT), and glutamate pyruvate transaminase (GPT) was performed using a veterinary hematology analyzer (Fuji DRI-Chem 3500s, Fujifilm, Tokyo, Japan) according to the manufacturer's protocols.

For hematologic toxicity test, vehicle- treated and NCT-80-treated mice were euthanized under isoflurane-induced deep anesthesia and blood collected by cardiac puncture. Blood samples were collected into tube containing EDTA. Analysis of WBC and RBC counts was performed with an automatic hematology analyzer (Advia 2120i, Siemens, Germany) according to the manufacturer's provided protocols.

### Immunohistochemistry

Sections derived from formalin-fixed and paraffin-embedded tumor tissues were deparaffinized by incubation overnight at 65°C followed by rehydration in sequential xylene and ethanol rinses. After incubation with hydrogen peroxide, the slides were washed with PBS and then incubated with 0.4% Triton X-100. After washing with PBS, the sections were incubated with blocking solution (Dako Protein Block, Dako, Glostrup, Denmark) for 30 min at room temperature. The sections were then incubated overnight at 4 °C with primary antibodies specific for pSTAT3 (Y705), STAT3, β-catenin, Sox2, PCNA, and cleaved caspase-3 (Cl-Cas3) (a 1:200 dilution). The sections were washed several times with PBS, incubated with the corresponding biotinylated secondary antibodies (a 1:500 dilution), and then again washed multiple times with PBS. After adding avidin-biotin complexes (Vector Laboratories, Burlingame, CA, USA), the sections were visualized using diaminobenzidine (DAB) detection reagent (Enzo Life Sciences) and cover-slipped using a mounting solution (Vector Laboratories).

### Molecular modeling

In silico study to evaluate the binding model of NCT-80 to the C-terminal domain of Hsp90 was carried out as described previously 25, 28. A docking modeling was conducted using the Tripos Sybyl-X 2.1 program in Windows 7 operating system, and the visualization of docking results were performed on Maestro Graphic User Interface in Schrödinger 2020-4. Molecular structure of ligand was prepared as mol2 format using the sketch module in Sybyl. Gasteiger-Hückel charges were assigned to all atoms of ligand, and minimized with the conjugate-gradient method with the convergence criterion of 0.001 kcal mol^-1^.Å^-1^. We used Surflex-Dock program for docking modeling of binding pose of NCT-80 in the C-terminal domain of human HSP90 (hHSP90). Our previously reported homology model of hHSP90:ATP complex 30 was used as a receptor for docking. The protomol, a computational description of the binding cavity at which putative ligands are aligned, was defined manually. Twenty amino acid residues adjacent to the ATP-binding site of hHSP90 were selected to define a protomol, and then it was generated with a threshold parameter of 0.50 and a bloat parameter of 0 Å. Docking was conducted with the default settings of Surflex-Dock GeomX, with generating 50 maximum poses per ligand and performing CScore (consensus score) calculations 31. Binding affinity of each pose of ligand was estimated by Surflex-Dock score (-log K_d_) which takes into account of hydrophobic, polar, repulsive, entropic, and salvation terms. The final docking model was selected by Surflex-Dock score, CScore, and visual inspection.

### Statistical analysis

Data are presented as the mean ± standard deviation (SD). Statistical significance was determined using a two-tailed Student's *t*-test or one-way ANOVA using GraphPad Prism 9 (GraphPad Software Inc., La Jolla, CA, USA). Statistical significance was set at *P* < 0.05.

## Results

### Akt- and ERK-mediated transcriptional upregulation of IL6 causes STAT3 activation, leading to resistance to Hsp90 inhibitors in NSCLC cells

**Figure 1:**
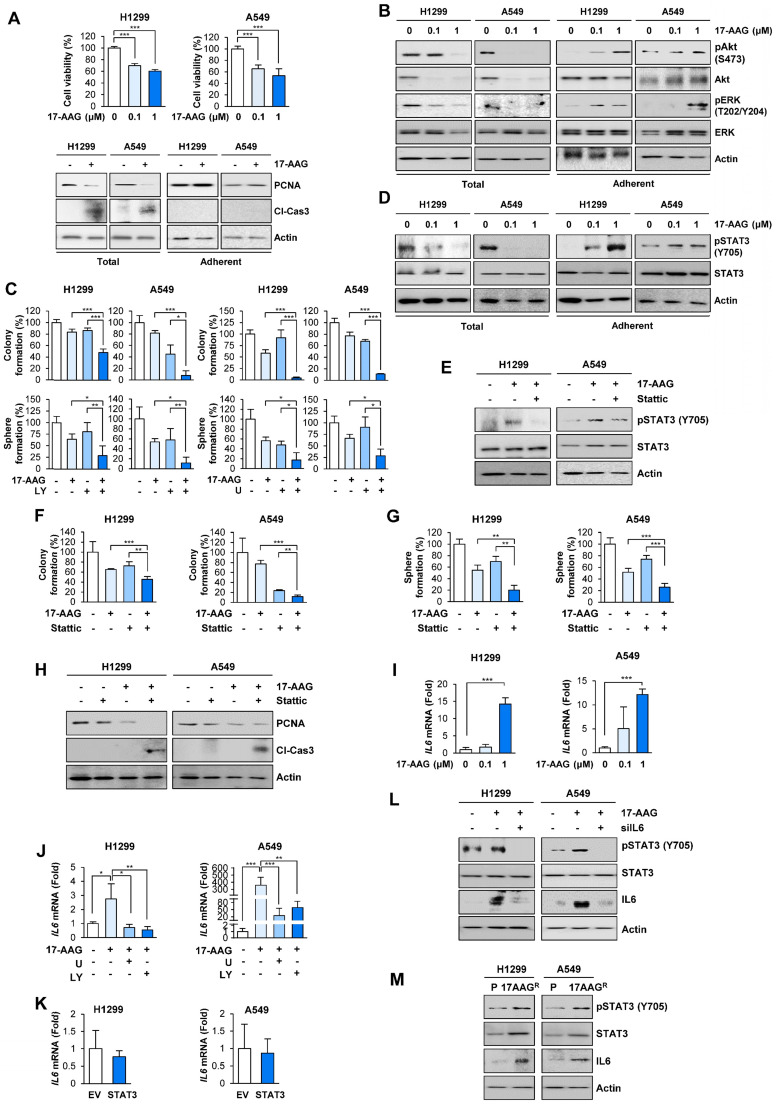
** Activation of ERK and Akt in surviving cells after blockade of Hsp90 for 5 days cause STAT3 activation through increasing *IL6* transcription, leading to cell survival and acquisition of CSC properties in NSCLC cells.** (**A, top**) MTT assay showing changes in cell viability by treatment with 17-AAG (0.1 and 1 μM) for 5 days. (**A, bottom**) Western blot (WB) analysis showing changes in levels of PCNA expression and cleavage of caspase-3 (Cl-Cas3) by treatment with 17-AAG (1 μM) for 5 days. (**B**) WB analysis showing Akt and ERK activation by treatment with 17-AAG for 5 days. (**C**) Changes in anchorage-dependent colony formation and sphere formation by treatment with 17-AAG (1 nM for H1299 cells; 0.1 nM for A549 cells) either alone or in combination with U0126 (U, 1 μM) or LY294002 (LY, 2 μM for H1299 cells; 0.5 μM for A549 cells) for 2 weeks. (**D, E**) WB analysis showing STAT3 activation by treatment with 17-AAG (1 μM) in the absence (**D, E**) or presence (**E**) of Stattic (1 μM) for 5 days. (**F, G**) Changes in anchorage-dependent colony formation (**F**) and sphere formation (**G**) by treatment with 17-AAG (1 nM for H1299 cells; 0.1 nM for A549 cells), either alone or in combination with Stattic (1 μM for H1299 cells; 0.1 μM for A549 cells), for 2 weeks. (**H**) WB analysis showing changes in levels of PCNA expression and cleavage of caspase-3 (Cl-Cas3) by treatment with 17-AAG (0.1 μM), either alone or in combination with Stattic (1 μM) for 5 days. (**I, J**) Real-time PCR analysis showing changes in the *IL6* mRNA expression by treatment with 17-AAG (**I:** 0.1 and 1 μM; **J:** 1 μM), either alone (**I**) or in combination with U0126 (U, 1 μM) or LY294002 (LY, 2 μM) (**J**), for 5 days. (**K**) Real-time PCR analysis showing modulation of *IL6* mRNA expression by overexpression of constitutively activated STAT3 (CA-STAT3). (**L**) WB analysis showing modulation of STAT3 activation and IL6 expression by treatment with 17-AAG (1 μM) with or without siRNA-mediated *IL6* silencing. (**M**) WB analysis showing modulation of STAT3 activation and IL6 expression in acquired 17-AAG-resistant (17AAG^R^) H1299 and A549 sublines compared with those in the corresponding parental (P) cells. The bars represent mean ± SD; *P < 0.05, **P < 0.01, and ***P < 0.001, as determined by a two-tailed Student's *t*-test (**A, I**), one-way ANOVA with Dunnett's post-hoc test (**C, F, G**) or one-way ANOVA with Tukey's post-hoc test (**J**) in comparison with the indicated group. Cl-Cas3: cleaved caspase-3.

Studies have shown that anticancer drug resistance emerges by the expansion of pre-existing resistant subclones or evolution of drug resistant cancer cells 32. To isolate NSCLC sublines that survive against blockade of the Hsp system, we treated two human NSCLC cell lines (H1299 and A549) with the Hsp90 inhibitor 17-allylamino-17-demethoxygeldanamycin (17-AAG, tanespimycin) and Hsp90 inhibitor-resistant NSCLC cell subpopulations. Treatment with 17-AAG for 5 days induced dose-dependent decrease in surviving cell population (**Figure 1A, top**). We postulated that the 'total (adherent and floating) cell' population after treatment with 17-AAG comprises drug-sensitive, drug-tolerant, and drug-resistant subclones while the 'adherent cell' population mainly comprises drug-tolerant and drug-resistant subclones. In fact, total [adherent (live) and floating (dead)] cell populations showed markedly decreased proliferating cell nuclear antigen (PCNA) and increased cleavage of caspase-3 expression (**Figure 1A, bottom left**). In contrast, the adherent (live) cell population exhibited minimal changes in the levels of PCNA and cleaved caspase-3 compared with vehicle-treated control cells (**Figure 1A, bottom right**). We further postulated that the 'adherent cell' populations may survive in the presence of anticancer therapeutics possibly through activation of signal transduction mechanisms that induce cell proliferation and/or survival. Indeed, the adherent cell population but not total cell population showed significantly increased activation (phosphorylation) of ERK and Akt, which play key roles in cell proliferation and survival (**Figure 1B**). Notably, treatment with inhibitors targeting MEK/ERK (U0126; U) or PI3K/Akt (LY294002; LY) enhanced the 17-AAG-mediated inhibition of anchorage-dependent colony formation and sphere formation, general properties of clonogenic cell survival and CSC-like phenotype, respectively 33, 34(**Figure 1C**).

Based on the previous observations of the JAK/STAT3 pathway activation in ganetespib-resistant cells 35 and role of STAT3 in anticancer drug resistance and acquisition of CSC properties 20, 21, we assessed whether STAT3 was implicated in the 17-AAG resistance. Indeed, the adherent subpopulations of H1299 and A549 cells after the 17-AAG treatment revealed markedly increased STAT3 activation (phosphorylation) unlike total cell populations (**Figure 1D**). Blockade of STAT3 by pharmacological inhibitor (Stattic) suppressed 17-AAG-induced STAT3 phosphorylation in the adherent cell population (**Figure 1E**). Moreover, compared to single treatments, combined treatment with Stattic and 17-AAG induced significantly greater decreases in anchorage-dependent colony formation (**Figure 1F**), sphere formation (**Figure 1G**), and PCNA expression along with increase in caspase-3 cleavage (**Figure 1H**). Therefore, the adherent cells could have been either tolerant or resistant to 17-AAG-induced apoptotic cell death by activating ERK, Akt, and STAT3.

We then investigated the mechanism underlying resistance to 17-AAG. Based on previous findings showing therapy-induced tumor repopulation via soluble factors 13, increase in IL6 expression through the RAF/MEK/ERK and PI3K/AKT/mTOR signaling pathways 36, 37, and Hsp90 blockade-caused secretion of IL6 35, 38, a ligand for IL6 receptor that activates STAT3, we speculated that activation of RAF/MEK/ERK and PI3K/AKT/mTOR signaling pathways after 17-AAG treatment caused transcriptional changes in IL6 expression, leading to STAT3 activation. Indeed, the adherent subpopulations after 17-AAG treatment revealed dose-dependent increase in *IL6* transcription (**Figure 1I**), and combination treatment with U0126 or LY294002 significantly suppressed 17-AAG-induced increase in *IL6* transcription (**Figure 1J**). *IL6* transcription in H1299 and A549 cells with enforced overexpression of constitutively activated STAT3 (CA-STAT3 39) remained unchanged (**Figure 1K**). In contrast, inhibition of IL6 by siRNAs suppressed the 17-AAG-induced activation of STAT3 and IL6 expression (**Figure 1L**). These findings suggested that upregulated IL6 expression through activation of RAF/MEK/ERK and PI3K/AKT/mTOR signaling pathways was responsible for the activation of STAT3.

To further confirm activation of the IL6/STAT3 pathway in 17-AAG-resistant cells, we further analyzed H1299 and A549 cell subpopulations that were subjected to 17-AAG exposure over two months, when no floating (dying) cells were observed. H1299 and A549 sublines that survived against the prolonged exposure to 17-AAG (H1299/AAG^R^ and A549/AAG^R^) revealed upregulated expression of IL6 and total and phosphorylated STAT3 compared with their corresponding parental cells (**Figure 1M**). These findings suggested that blockade of Hsp90 induces compensatory activation of ERK and Akt, causing *IL6* transcriptional upregulation and STAT3 activation, resulting in anticancer drug resistance.

### STAT3 activation following Hsp90 blockade activates Wnt signaling via transcriptional upregulation of Wnt ligands, resulting in expansion of surviving NSCLC cells

**Figure 2:**
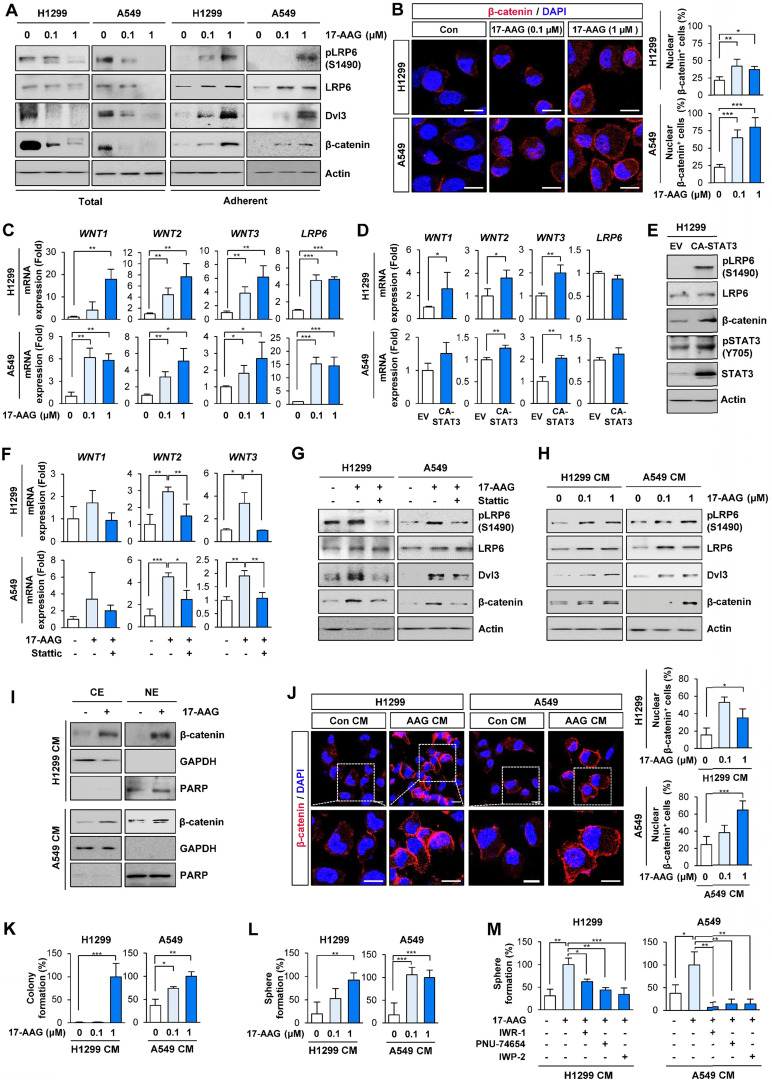
** Chronic Hsp90 blockade induces the expression of Wnt ligands and autocrine and/or paracrine activation of the Wnt signaling pathway, causing enrichment of cancer stem cells (CSCs).** (**A**) Western blot (WB) analysis showing changes in activation of the Wnt signaling pathway by treatment with 17-AAG for 5 days. (**B**) Immunofluorescence (IF) analysis showing changes in nuclear translocation of β-catenin. Representative images are shown. Scale bars: 20 μm. (**C**) Real-time PCR analysis showing changes in mRNA expression of Wnt ligands and LRP6 by treatment with 17-AAG for 5 days. (**D**) Real-time PCR analysis showing changes in mRNA expression of Wnt ligands and LRP6 by overexpression of constitutively active form of STAT3 (CA-STAT3). (**E**) WB analysis showing changes in the activation of the Wnt signaling cascade and STAT3 by overexpression of CA-STAT3. (**F, G**) Real-time PCR (**F**) and WB (**G**) analyses showing changes in mRNA expression of Wnt ligands (**F**) and Wnt signaling activation (**G**) by treatment with 17-AAG (1 nM for H1299 cells; 0.1 nM for A549 cells) either alone or in combination with Stattic (1 μM for H1299 cells; 0.1 μM for A549 cells) for 5 days. (**H-L**) H1299 and A549 cells (> 60% confluency) were treated with vehicle or 17-AAG (0.1 or 1 μM) for 5 days. The media was replaced with fresh serum-free media and cells were incubated for 1 day. Conditioned medium (CM) was collected from these donor cells and then used to treat naïve H1299 and A549 cells (recipient cells). (**H-J**) Determination of changes in activation of the Wnt signaling pathway (**H**) and nuclear translocation of β-catenin (**I, J**) in recipient cells by WB (**H, I**) and IF (**J**) analyses. (**J**) Representative images are shown. Com CM: CM derived from vehicle-treated control cells. AAG CM: CM derived from 17-AAG (1 μM)-treated cells. Scale bars: 20 μm. (**K, L**) Changes in anchorage-dependent colony formation (**K**) and sphere formation (**L**) of recipient cells by treatment with donor cells-derived CM. (**M**) Changes in sphere formation after treatment with donor cells-derived CM. H1299 and A549 cells (> 60% confluency) were treated with vehicle or 17-AAG in the presence or absence of Wnt inhibitors (10 μM IWR-1, 10 μM PNU-74654, or 5 μM IWP-2) for 5 days. Cells were washed twice with PBS, and the media was then replaced with fresh serum-free media and cells were incubated for 1 day. CM were collected and then used for further experiments. For sphere formation assay, naïve H1299 and A549 cells were treated with CM, either alone or in presence of Wnt inhibitors (10 μM IWR-1 or 10 μM PNU-74654), for 2 weeks. The bars represent mean ± SD; *P < 0.05, **P < 0.01, and ***P < 0.001, as determined by a two-tailed Student's *t*-test (**B, C, D, J, K, L**) or one-way ANOVA with Tukey's post-hoc test (**F, M**) in comparison with the indicated group. Con: control. CM: conditioned medium. CE: cytoplasmic extracts. NE: nuclear extracts. EV: empty vector. CA-STAT3: constitutively active STAT3.

Based on previous studies demonstrating the crosstalk between Wnt and STAT3 signaling pathways 40, we next investigated whether Hsp90 blockade by 17-AAG treatment activated Wnt signaling. Indeed, 17-AAG-treated adherent H1299 and A549 cells, but not total cell populations, showed significantly increased expression of total and phosphorylated LRP (pLRP), Dvl3, and β-catenin protein (**Figure 2A**). Immunofluorescence (IF) analysis of the 17-AAG-treated NSCLC cells revealed increased nuclear translocation of β-catenin, an indicator of Wnt signaling pathway activation (**Figure 2B**).

We then investigated the role of the Wnt signaling in resistance to 17-AAG utilizing the 'adherent cell' population rather than the 'total cell' population. Notably, H1299 and A549 cells that survived 5 days of 17-AAG treatment exhibited markedly increased transcription of Wnt ligands (*WNT1, WNT2,* and* WNT3*) and LDL Receptor Related Protein 6 (LRP6) compared with control cells (**Figure 2C**). The enforced overexpression of CA-STAT3 resulted in *WNT1*, *WNT2* and* WNT3* mRNA (**Figure 2D**) and pLRP6 and β-catenin protein expression (**Figure 2E**). Moreover, STAT3 inhibition by treatment with Stattic suppressed 17-AAG-induced increased expression of Wnt ligand mRNA (**Figure 2F**) and pLRP6, Dvl3, and β-catenin protein (**Figure 2G**). Meanwhile, *LRP6* mRNA expression showed minimal changes on CA-STAT3 overexpression (**Figure 2D**), indicating 17-AAG-induced increase in *LRP6* expression might be mediated through a STAT3-independent mechanism. We further confirmed significant increases in *WNT1*, *WNT2* and* WNT3* mRNA in 17-AAG-resistant H1299 and A549 cells (H1299/AAG^R^ and A549/AAG^R^ cells) compared with those in their corresponding parental cells (**Figure S1**).

Based on the STAT3-mediated soluble factor production from cancer and stromal cells 18, 41, the conditioned media (CM) from H1299 and A549 cells that survived 5 days of 17-AAG treatment were added to naïve NSCLC cells. The CM-treated recipient cells exhibited increased expression of pLRP, Dvl3 and β-catenin proteins (**Figure 2H**). Western blot (**Figure 2I**) and IF (**Figure 2J**) analyses showed increased nuclear localization of β-catenin in the CM-treated recipient cells. Moreover, recipient cells incubated with CM from the 17-AAG-treated NSCLC cells showed significantly increased capacities for anchorage-dependent colony formation (**Figure 2K**) and sphere formation (**Figure 2L**). The CM from 17-AAG-treated donor cells no longer stimulated the sphere forming capacities of H1299 and A549 recipient cells when incubated with Wnt signaling inhibitors, including IWR-1 [a tankyrase inhibitor that suppress the Wnt signaling pathway by disrupting tankyrase-mediated poly(ADP‐ribosyl)ation of Axin2] 42, IWP-2 [a Wnt antagonist preventing Wnt ligand palmitoylation by targeting the membrane-bound O-acyltransferase porcupine (Porcn)] 43, or PNU-74654 [a Wnt inhibitor targeting the interaction between β-catenin and Tcf4] 44 (**Figure 2M**). These findings collectively suggested the expansion of drug-tolerant or drug-resistant cells following Hsp90 blockade through STAT3-mediated Wnt signaling activation via transcriptional upregulation of Wnt ligands.

### NCT-80 is a potential antitumor agent that disrupts viability, migration, and Wnt signaling activation in NSCLC cells without overt toxicity to normal cells

We attempted to develop novel Hsp90 inhibitors that have a different mode of action compared to the Hsp90 inhibitors targeting N-terminal ATP-binding pocket. We have demonstrated that deguelin, a naturally occurring rotenoid, displays potent anticancer activities by disrupting ATP binding to Hsp90 45. We have also synthesized a variety of deguelin analogs that block the C-terminal ATP-binding pocket 11, 25. In the present study, we synthesized 14 chemical compounds based on a B- and C-ring truncation of deguelin (**Table 1**). These chemicals contained a common chemical moiety of two aromatic rings (5-methoxy-2,2-dimethyl-2H-chromene and 1-methoxy phenyl rings) linked by an amide bond in which a variety of polar side chains as solubilizing groups were incorporated at the 2-position of the phenyl ring. Among them, NCT-88 is a prototype inhibitor that has a 2-methoxy group. As a solubilizing group, six compounds have a 2-aminoalkyloxy group (NCT-55, 66, 69, 82, 96, and 101), four compounds have a 2-piperidinylalkyloxy group (NCT-375, 385, 394, and 407) and the remaining three compounds possess a 2-pyridylalkyloxy group (NCT-80, 377, and 383) (**Table 1**).

**Figure 3:**
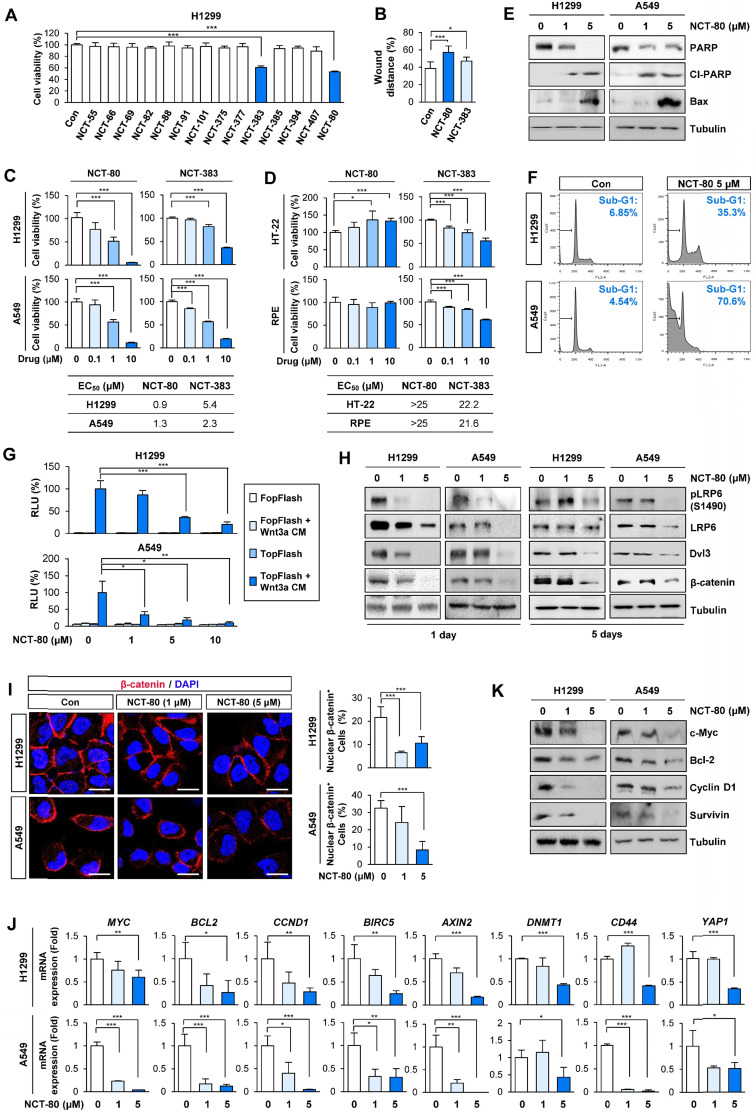
** NCT-80 suppresses migration, Wnt signaling activity, and viability of lung cancer cells with minimal toxicity to normal cells.** (**A-D**) Cells were treated with 1 μM test compounds (**A, B**) or increasing concentrations of NCT-80 or NCT-383 (**C, D**) for 3 days. (**A, C, D**) Determination of cell viability using an MTT assay. (**B**) Determination of the effects of NCT-80 and its derivatives on cell migration using a wound-healing assay. (**E, F**) Determination of effects of NCT-80 on induction of apoptosis by Western blot (WB) analysis (**E**) and flow cytometry (**F**). (**G**) Determination of the effects of NCT-80 on β-catenin/Tcf-mediated transcriptional activity using a luciferase reporter assay. (**H**) Determination of activation of the Wnt signaling pathway in H1299 and A549 cells treated with NCT-80 for 1 or 5 days by WB analysis. (**I**) Immunofluorescence analysis showing nuclear translocation of β-catenin in H1299 and A549 cells treated with NCT-80 for 1 day. Representative images are shown. Scale bars: 20 μm. (**J, K**) Real-time PCR (**J**) and WB (**K**) analyses showing regulation of mRNA and protein expression of Wnt target genes in cells treated with NCT-80. The bars represent mean ± SD; *P < 0.05, **P < 0.01, and ***P < 0.001, as determined by a two-tailed Student's *t*-test in comparison with the indicated group. Con: control. Cl-PARP: cleaved poly-(ADP-ribose) polymerase. RLU: relative luciferase unit.

We evaluated the compounds for inhibitory effects on H1299 cell viability at a concentration of 1 μM. Among 14 compounds, NCT-80 and NCT-383, possessing a pyridine group in the solubilizing side chain, displayed the most potent inhibitory effect on H1299 cell viability (**Figure 3A**). We subsequently evaluated these compounds for their effects on cell migration. Wound-healing assay in H1299 cells revealed that NCT-80 exhibited superior inhibitory effects than NCT-383 on the migratory ability (**Figure 3B**). We next evaluated the effects of NCT-383 and NCT-80 on NSCLC cell viability. NCT-80 displayed greater dose-dependent inhibitory effects on viability of H1299 and A549 cells than did NCT-383 (**Figure 3C**). NCT-383 revealed more potent cytotoxicity in normal cells derived from mouse hippocampus (HT-22) and human retinal pigmental epithelium than did NCT-80 (**Figure 3D**). Notably, the NCT-80 treatment for 72h induced apoptosis in H1299 and A549 NSCLC cells, as measured by decreases in un-cleaved PARP levels, upregulation of cleaved PARP (Cl-PARP) and Bax expression (**Figure 3E**), and increases in sub-G1 population (**Figure 3F**). NCT-80 inhibited the Wnt3a-induced β-catenin/Tcf transcriptional activity in a dose-dependent manner (**Figure 3G**).

Importantly, Wnt signaling remained suppressed after 5 days of NCT-80 treatment, as measured by the expressions of phosphorylated LRP6 (pLRP6), Dvl3, and β-catenin (**Figure 3H**). The NCT-80-treated cells revealed attenuated nuclear localization of β-catenin (**Figure 3I**); reductions in the expression of Wnt signaling target genes (**Figure 3J**); and representative proteins encoded by the target genes, including cyclin D1, c-myc, Bcl-2, and survivin (**Figure 3K**). Together, these findings identify NCT-80 as a potential Hsp90-targeting antitumor agent that disturbs Wnt signaling in NSCLC cells without overt toxicity to normal cells.

### NCT-80 inhibits STAT3 activation by disrupting the interaction between Hsp90 and STAT3

**Figure 4:**
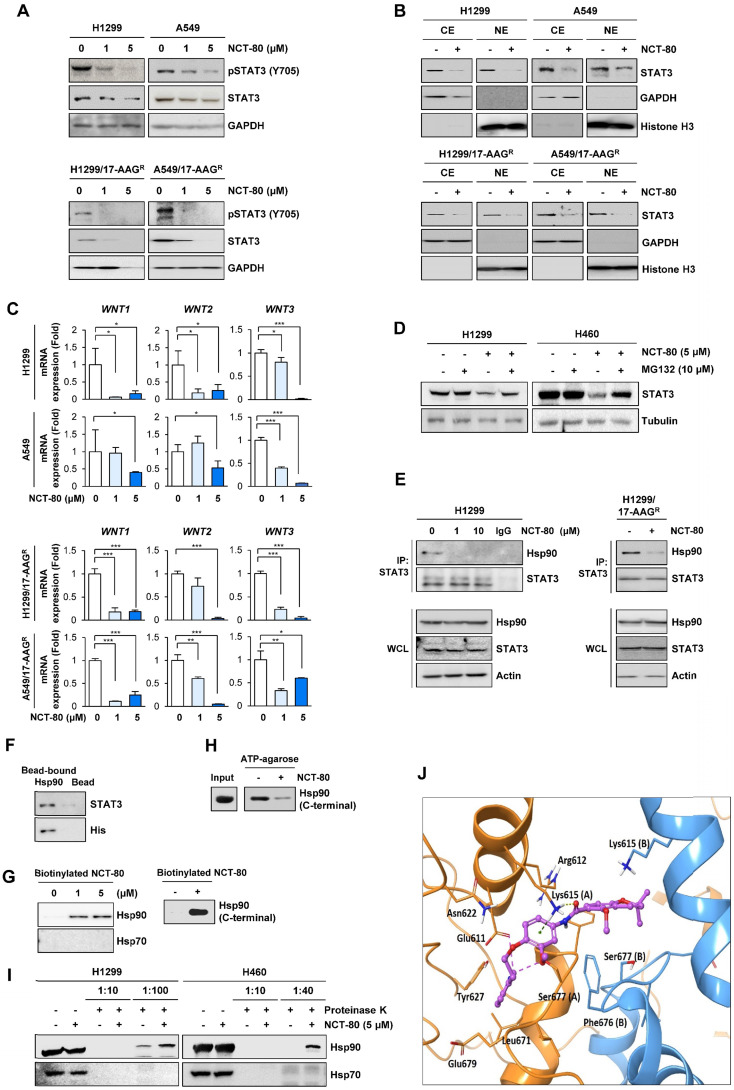
** NCT-80 inhibits the interaction between Hsp90 and STAT3 by binding to C-terminal domain of Hsp90 and disrupting Hsp90 function.** (**A**) Western blot (WB) analysis showing regulation of total or phosphorylated STAT3 expression in H1299 and A549 cells (**top**) or those resistant to 17-AAG (17AAG^R^, **bottom**) by treatment with NCT-80 for 5 days. (**B**) WB analysis showing regulation of nuclear translocation of STAT3 in H1299 and A549 cells (**top**) or those resistant to 17-AAG (17AAG^R^, **bottom**) by treatment with NCT-80 for 1 day. (**C**) Real-time PCR analysis showing regulation of mRNA expression of Wnt ligands in H1299 and A549 cells (**top**) or those resistant to 17-AAG (17AAG^R^, **bottom**) by treatment with NCT-80 for 5 days. (**D**) Determination the effect of NCT-80 on proteasomal degradation of STAT3 by WB analysis. Vehicle (DMSO)- or NCT-80-treated cells were further treated with MG132 (10 μM) for 6 h. (**E, F**) Determination of effects of NCT-80 on the interaction between endogenous Hsp90 and STAT3 (**E**) and the interaction between recombinant Hsp90 and endogenous STAT3 (**F**) by immunoprecipitation (**E**) and the pull-down (**F**) assays. (**G**) Determination of binding of NCT-80 to full-length (**left**) or the C-terminal domain (**right**) of Hsp90 by the pull-down assay using biotinylated NCT-80. (**H**) Pull-down assay using ATP-agarose showing effect of NCT-80 (5 μM) on the ATP binding to the C-terminal domain of Hsp90. (**I**) Determination of the binding of NCT-80 to full-length Hsp90 or Hsp70 by DARTS. (**J**) A docking model of NCT-80 in the ATP-binding pocket of hHsp90. Two chains of hHsp90 are illustrated in orange (Chain A) and blue ribbon (Chain B), respectively. The drug molecule is rendered in purple ball-and-stick, omitting hydrogen atoms for clarity. Intermolecular interactions between drug and protein are shown as dotted lines. The bars represent mean ± SD; *P < 0.05, **P < 0.01, and ***P < 0.001, as determined by a two-tailed Student's *t*-test in comparison with the indicated control. CE: cytoplasmic extracts. NE: nuclear extracts. WCL: whole cell lysates. IP: immunoprecipitation.

We next assessed whether NCT-80 regulated Wnt signaling by regulating STAT3 activity. To determine the effect of NCT-80 on nuclear translocalization of STAT3, H1299 and A549 cells were treated NCT-80 for 24 h, when the drug induced no cytotoxicity in the cells. Treatment with NCT-80 dose-dependently suppressed the expression of total and phosphorylated forms of STAT3 (**Figure 4A**) and nuclear translocation of STAT3 (**Figure 4B**) in H1299, A549, and their 17-AAG-resistant sublines (H1299/AAG^R^ and A549/AAG^R^). Moreover, treatment with NCT-80 significantly suppressed the expression of *WNT1*, *WNT2*, and *WNT3* in these cells (**Figure 4C**). Hence, the inhibitory effect of NCT-80 on the expression of Wnt ligands was associated with STAT3 inactivation. We next investigated how NCT-80 regulated STAT3 activation. Because STAT3 is a client of the Hsp90 system 46 and NCT-80 decreased total STAT3 protein level, we speculated that NCT-80 could have induced ubiquitination-mediated degradation of the STAT3 protein. Indeed, treatment with the proteasome inhibitor MG132 markedly restored STAT3 protein levels in NCT-80-treated NSCLC cells (**Figure 4D**). Immunoprecipitation (**Figure 4E**) and pull-down (**Figure 4F**) assays revealed that the interaction between Hsp90 and STAT3 was markedly suppressed by treatment with NCT-80. We next analyzed whether NCT-80 disrupted STAT3 protein stability by directly binding to Hsp90. Indeed, biotinylated NCT-80 displayed binding capacity to Hsp90, especially C-terminal Hsp90 (**Figure 4G**). NCT-80 was also able to inhibit ATP binding to Hsp90 C-terminal domain (**Figure 4H**). Drug affinity responsive target stability (DARTS) assay displayed that NCT-80 protected Hsp90 from protease-mediated proteolysis, confirming the direct binding of NCT-80 to Hsp90 (**Figure 4I**). Conversely, Hsp70 exhibited no binding activity to NCT-80 (**Figure 4G, 4I**).

We carried out a molecular docking study to determine the binding mode of NCT-80 in Hsp90 C-terminal domain using the homology model of open conformation of human Hsp90 homodimer 28, 47. NCT-80 fits well in the C-terminal domain of Hsp90. The alpha H on pyridine ring of NCT-80 forms weak hydrogen bonds both with the side-chain carboxyl group of Glu611 and hydroxyl group of Ser677 in the C-terminal ATP-binding pocket (**Figure 4J**). In addition, NCT-80 is involved in two interactions with the protonated side-chain amino group of Lys615 in the chain A of hHsp90: π-cation interaction formed by the central benzene ring, and hydrogen bond by carbonyl oxygen of amide linker of NCT-80. These results suggested that NCT-80 binds to the C-terminal ATP-binding pocket of Hsp90 and stabilizes the open conformation of Hsp90 homodimer, thereby inhibiting Hsp90 function.

### NCT-80 suppresses NSCLC cell viablity and migration occurred by disrupting epithelial-mesenchymal transition (EMT)

**Figure 5:**
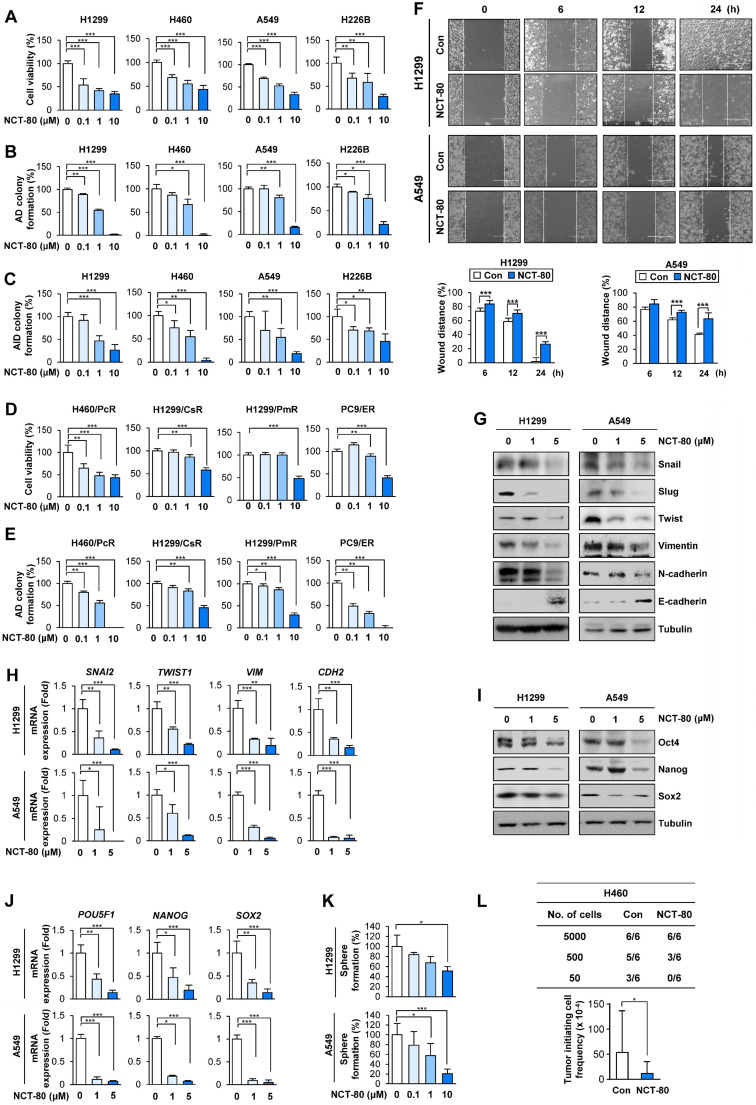
** NCT-80 inhibits the viability, migration, and CSC-like properties in NSCLC by inhibiting expression of EMT and stemness-associated markers.** (**A-E**) Determination of effects of NCT-80 on viability (**A, D**), anchorage-dependent (AD) colony formation (**B, E**), and anchorage-independent (AID) colony formation (**C**) of NSCLC cells (**A-C**) and those resistant to anticancer agents [paclitaxel-resistant H460 (H460/PcR), cisplatin-resistant H1299 (H1299/CsR), pemetrexed-resistant H1299 (H1299/PmR), and erlotinib-resistant PC9 (PC9/ER) cells] (**D, E**) using an MTT assay (**A, D**), anchorage-dependent colony formation assay (**B, E**), and soft agar colony formation assay (**C**). (**F**) Wound-healing assay showing effects of NCT-80 (5 μM) on migration of H1299 and A549 cells. (**G, H**) Western blot (WB) (**G**) and real-time PCR (**H**) analyses showing effects of NCT-80 on expression of EMT-associated markers in H1299 and A549 cells. (**I, J**) WB (**I**) and real-time PCR (**J**) analyses showing effects of NCT-80 on expression of stemness markers in H1299 and A549 cells. (**K**) Sphere formation assay showing effects of NCT-80 on sphere formation of H1299 and A549 cells (**L**) Limiting dilution assay showing effect of NCT-80 on tumorigenicity. ELDA determining tumor initiating cell frequency (**L, bottom**). The bars represent mean ± SD; *P < 0.05, **P < 0.01, and ***P < 0.001, as determined by a two-tailed Student's *t*-test in comparison with the indicated control. Con: control. AD: anchorage-dependent. AID: anchorage-independent.

We evaluated the therapeutic efficacy of NCT-80 in various types of NSCLC cells. NCT-80 significantly inhibited NSCLC cell viability (**Figure 5A**) and colony formation under anchorage-dependent (**Figure 5B**) and anchorage-independent (**Figure 5C**) culture conditions in a dose-dependent manner. Considering the clinical utility of anticancer therapy for patients who progressed upon chemotherapy and molecular targeted therapy and recent studies showing Hsp90 involvement in the resistance to various antitumor agents including chemotherapy and molecular targeted therapy 48, we additionally evaluated the effect of NCT-80 on the viability and colony-forming capacities of several NSCLC cell sublines that are resistant to chemotherapeutics (designated '/R'), including paclitaxel (H460/PcR), cisplatin (H1299/CsR), and pemetrexed (H1299/PmR), and the EGFR TKI erlotinib (PC9/ER), which were established in our previous studies 25-27. NCT-80 exhibited comparable inhibitory effects on the viability (**Figure 5D**) and colony-forming capacities (**Figure 5E**) of these drug resistant cells.

Given the roles of STAT3 and Wnt signaling in EMT 49 and the impact of EMT on migration and invasion of cancer cells 49, we evaluated the effect of NCT-80 on the migration and expression of EMT-related markers of NSCLC cells. Treatment with NCT-80 significantly suppressed wound closure resulting from H1299 and A549 NSCLC cell migration, indicating the inhibitory effects of NCT-80 on NSCLC cell migration (**Figure 5F**). NCT-80 also demonstrated the ability to control protein (**Figure 5G**) and mRNA (**Figure 5H**) levels of EMT markers (i.e., decreases in Snail, Slug, Twist, Vimentin, and N-Cadherin and increase in E-cadherin) in NSCLC cells.

Since STAT3 and Wnt signaling play key roles in CSC-like properties 15, 20, we next explored the effects of NCT-80 on CSC-like phenotypes of NSCLC cells. NCT-80 suppressed protein (**Figure 5I**) and mRNA (**Figure 5J**) expression of well-known stemness markers, such as Oct4, Nanog, and Sox2 50. Dose-dependent inhibitory effects of NCT-80 on the sphere-forming ability of NSCLC cells were also observed (**Figure 5K**). Consistent with these *in vitro* results, NCT-80 treatment also reduced the tumorigenic ability of NSCLC cells compared with control (**Figure 5L**). Extreme limiting dilution analysis (ELDA) displayed reduction in the tumor initiating cell frequency by treatment with NCT-80 (**Figure 5L, bottom**). These results collectively indicated that NCT-80 has potent antitumor activities in NSCLC cells.

### NCT-80 displays potent antitumor activities in NSCLC cells *in vivo* and potentiates anticancer effects of anticancer therapeutics without overt toxicity

**Figure 6:**
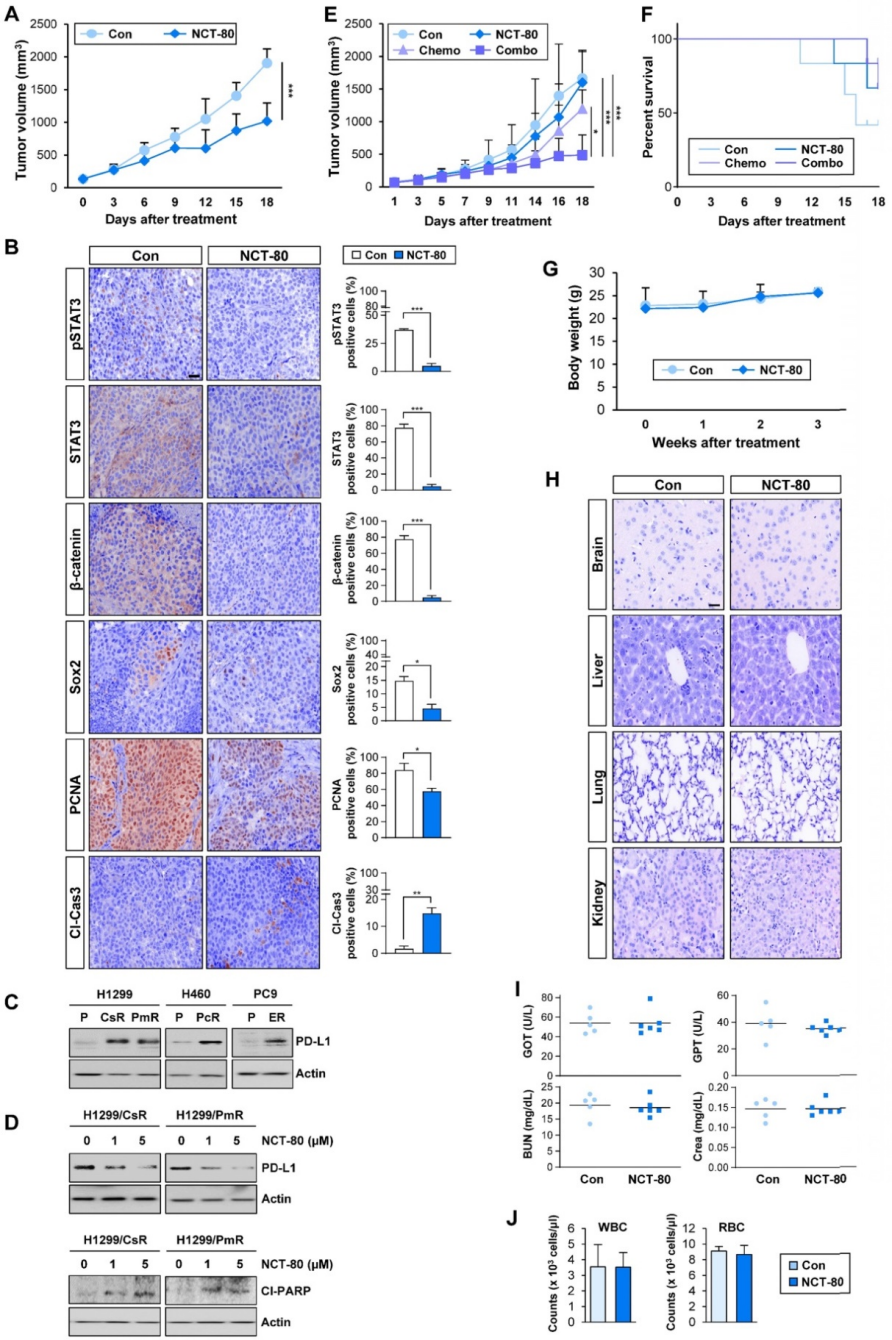
** NCT-80 displays antitumor activity *in vivo* and enhances antitumor effect of chemotherapeutic agents with no overt toxicity.** (**A**) Effect of NCT-80 (50 mg/kg) on growth of patient-derived xenograft tumors (*n* = 6 per group). (**B**) Immunohistochemistry analyses showing the effect of NCT-80 on expression of pSTAT3 (Y705), STAT3, β-catenin, Sox2, PCNA, and cleaved caspase-3 (Cl-Cas3). Scale bar: 100 μm. (**C, D**) Western blot analysis showing the level of PD-L1 expression in chemoresistant cells (**C**) and effect of NCT-80 on the expression of PD-L1 (**D, top**) and PARP cleavage (Cl-PARP) (**D, bottom**). (**E, F**) Effect of NCT-80 (50 mg/kg), either alone or in combination with chemotherapeutic agents [Chemo: paclitaxel (Pc, 20 mg/kg) and cisplatin (Cs, 3 mg/kg) in combination], on the growth of LLC-Luc allograft tumors (**E**) and survival of mice (**F**) (*n* = 6 per group). (**G**) Changes in body weight of vehicle-treated or NCT-80-treated mice (*n* = 6 per group). (**H**) Changes in histological features of major organs (brain, liver, kidney, and lung) in vehicle-treated or NCT-80-treated mice. Scale bar: 100 μm. (**I**) Changes in serum levels of GOT, GPT, BUN, and creatinine (Crea) of NCT-80-treated mice compared with those of vehicle-treated mice. (**J**) Changes in the number of white blood cells (WBC) and red blood cells (RBC) in blood of NCT-80-treated mice compared with those of vehicle-treated mice. The bars represent mean ± SD; *P < 0.05, **P < 0.01, and ***P < 0.001, as determined by a two-tailed Student's *t*-test (**A, B**) or one-way ANOVA with Dunnett's post-hoc test (**E**) in comparison with the indicated control. Con: control. CsR: cisplatin-resistant cells. PmR: pemetrexed-resistant cells. PcR: paclitaxel-resistant cells. ER: erlotinib-resistant cells. Cl-PARP: cleaved poly-(ADP-ribose) polymerase.

The antitumor effect of NCT-80 was evaluated in a NSCLC patient-derived xenograft tumor model. Consistent with *in vitro* results, NCT-80 significantly suppressed the growth of patient-derived xenograft (PDX) tumors (**Figure 6A**). Immunohistochemistry (IHC) analysis revealed downregulation of total and phosphorylated STAT3, β-catenin, Sox2, and PCNA and increased expression of the cleaved form of caspase-3 (Cl-Cas3) in tumors from NCT-80-treated mice (**Figure 6B**). Thus, NCT-80 appeared to inhibit STAT3/Wnt signaling-mediated signaling *in vivo* in the PDX tumors, causing apoptosis and thereby suppressing tumor growth.

Recent studies have demonstrated the role of PD-L1 in evading immunosurveillance by causing dysfunction of cytotoxic T cells and resistance to anticancer therapeutics by intrinsically activating signaling pathways that promote cancer cell survival 51. Of note, some transcription factors involved in the regulation of PD-L1 expression such as hypoxia inducible factor-1 alpha (HIF-1α) and STAT3 52, 53 are Hsp90 client proteins 46. Since chemotherapeutic drugs and epidermal growth factor receptor (EGFR) tyrosine kinase inhibitor (TKI) were found to promote immune escape of NSCLC cells by upregulating PD-L1 expression 54, 55, we hypothesized that NCT-80-mediated Hsp90 blockade may enhance antitumor activities of chemotherapy and EGFR-targeting anticancer therapy. Indeed, H460/PcR, H1299/CsR, H1299/PmR, and PC9/ER 25-27 cells displayed upregulated PD-L1 expression (**Figure 6C**), and treatment with NCT-80 considerably suppressed PD-L1 expression and induced PARP cleavage (**Figure 6D**). More importantly, combinatorial treatment with NCT-80 and chemotherapeutic agents (paclitaxel and cisplatin in combination, Pc/Cs) more effectively suppressed the growth of LLC-Luc allograft tumors (**Figure 6E**). During the drug treatment, mice treated with chemotherapy (Pc/Cs) and NCT-80, either alone or in combination, displayed improved survival compared with vehicle-treated control mice (**Figure 6F**). These findings indicated the potential of NCT-80 to suppress immune escape in NSCLC by downregulating PD-L1 expression.

We determined the potential toxicity of NCT-80. NCT-80 showed minimal cytotoxicity in normal cells derived from mouse hippocampus (HT-22) and human retinal pigmental epithelium (**Figure 3D**). During drug treatment *in vivo*, mice administered with NCT-80 exhibited minimal changes in body weight (**Figure 6G**). Histological analysis of H&E-stained tissues from various major organs including the brain, liver, kidney, and lung of NCT-80-treated mice also indicated no detectable changes compared with that of the control mice (**Figure 6H**). To further analyze the potential toxicity of NCT-80, FVB/N mice were administered vehicle (corn oil) or NCT-80 (50 mg/kg) by oral gavage every day for two weeks. NCT-80-treated mice revealed minimal changes in the serum levels of glutamate oxaloacetate transaminase (GOT) and glutamate pyruvate transaminase (GPT) (i.e., liver function parameters 56), blood urea nitrogen (BUN) and creatinine (**Figure 6I**) (i.e., renal function parameters 56), and the red blood cell (RBC) and white blood cell (WBC) counts (i.e., parameters for hematological toxicity 56) (**Figure 6J**) compared with vehicle-treated control mice. Taken together, these results suggested that NCT-80 exerts antitumor effects with minimal toxicity.

## Discussion

At the molecular level, Hsp90 targeting drugs have been developed as effective anticancer remedies. In this study, we showed the emergence of 17-AAG resistance in NSCLC cells through compensatory activation of Akt- and ERK-mediated sequential events; this includes the transcriptional upregulation of *IL6* and subsequent activation of STAT3, which in turn stimulates transcription of Wnt ligands and activation of Wnt signaling pathway. We further identified NCT-80 as a novel Hsp90 inhibitor that significantly inhibits viability, colony formation, migration, and CSC-like phenotypes in NSCLC cells and PDX tumor growth in mice without overt toxicity. Furthermore, NCT-80 inhibited PD-L1 expression in NSCLC cells that acquired resistance to chemotherapeutic drugs or an EGFR TKI and enhanced the antitumor effects of chemotherapeutic agents. Mechanistically, NCT-80 directly interacts with the C-terminal domain of Hsp90, disrupts the interaction between Hsp90 and STAT3 and protein stability of STAT3, and suppresses STAT3-mediated activation of Wnt signaling. These results support the use of NCT-80 as a novel Hsp90-targeting antitumor drug.

Considering the crucial role of Hsp system in the stabilization and maturation of various client oncoproteins and deregulated overexpression of Hsp90 in multiple types of cancers, the Hsp system has been considered as an effective target for anticancer therapy. Widespread efforts have been made to develop potent anticancer drugs targeting the system; several Hsp90 inhibitors, especially those targeting its N-terminal ATP-binding pocket, have been developed and evaluated in a number of preclinical and clinical studies 57. However, none have been approved to date for clinically use due to limited efficacy, severe toxicity, and/or drug resistance 11, 58.

Hence, we aimed to understand the bypass molecular mechanisms that cause resistance to currently available Hsp90 inhibitors and develop safe and efficacious drugs that target Hsp system without inducing drug resistance. Several mechanisms, including the induction of a compensatory heat shock response, addiction to RAF/MEK/ERK and PI3K/AKT/mTOR signaling, and activation of the Jak-STAT3 pathway, have been implicated in the resistance to Hsp90 inhibitors 59. Consistent with these findings, the present study results revealed the activation of STAT3, Akt, and ERK and transcriptional upregulation of *IL6* in NSCLC cells that survived 17-AAG treatment. Our results indicate that Akt, ERK, and STAT3 play essential roles in cell survival and CSC-like phenotypes 20, 22. Moreover, the Hsp90 inhibitor ganetespib was found to induce addiction to ERK and Akt signaling and upregulated production of IL6 35, 60, 61. Additionally, ERK and Akt signaling have been implicated in *IL6* transcription 36, 37. Based on the aforementioned findings, we hypothesized that STAT3 may act as a downstream effector of autocrine IL6 signaling and a key driver of the resistance to Hsp90 inhibitors, especially those that block the N-terminal ATP-binding pocket of Hsp90. Our data is consistent with those from validation studies that have revealed that pharmacological approaches targeting Akt, ERK, and STAT3 apparently suppress STAT3 activation and clonogenic cell survival and CSC properties in the 17-AAG-resistant subpopulation.

The next important question was how STAT3 activation results in resistance to the Hsp90 inhibitors. We describe herein that Hsp90 inhibitors targeting the N-terminal ATP-binding pocket cause STAT3-mediated transcription of Wnt ligands. Although the Wnt pathway is a key developmental signaling pathway that controls the maintenance and differentiation of stem cells 14, aberrantly activated Wnt signaling is involved in various pathological conditions, including cancer 14. The Wnt signaling pathway is also associated with the regulation of CSCs and plays an important role in tumor heterogeneity and plasticity, resulting in the acquisition of anticancer drug resistance and recurrent tumor formation 15. Indeed, the suppression of Wnt signaling effectively suppressed the CSC-like phenotypes of 17-AAG-resistant subpopulation. Therefore, Wnt signaling activation may be responsible for the expansion of pre-existing Hsp90 inhibitor-resistant subclones or evolution of drug-resistant cells. Previous studies have demonstrated positive regulation of Wnt signaling by Hsp90 via direct interaction of Hsp90 and LRP5 62 and the destabilization of β-catenin by pharmacological inhibition of Hsp90 63. The reciprocal regulation between STAT3 and Wnt signaling has been also demonstrated in previous studies. For example, STAT3 positively regulated β-catenin by increasing the transcription of *CTNNB1*, the gene encoding β-catenin, or Wnt ligand genes, such as *WNT3A* 64. Conversely, the Wnt signaling pathway upregulates *STAT3* mRNA expression in murine embryonic stem cells 65. However, the role of Wnt signaling pathway in resistance to Hsp90 inhibitors has been poorly investigated. Our findings suggest a scenario in which NSCLC cells acquire resistance to Hsp90 inhibitor via activation of IL-6/STAT3 signaling and subsequently Wnt signaling pathway.

We have, therefore, endeavored to develop novel safe and efficacious Hsp90-targeting anticancer interventions that overcome Wnt signaling-mediated drug resistance. We hypothesized that Hsp90 C-terminal inhibitors may offer an efficacious cancer therapy based on the following previous findings: 1) the presence of a second C-terminal ATP-binding site in Hsp90 25; 2) the role of the C-terminal ATP-binding pocket in Hsp90 dimerization, which is critical for its chaperone function and interaction with cochaperones; and 3) lack of cytoprotective Hsp70 induction by Hsp90 C-terminal inhibitors 6. NCT-80 was found to be one such lead candidate. Our biochemical and in silico studies show that NCT-80 disrupts the conformation of Hsp90 by interacting with the ATP-binding pocket of Hsp90 located in the C-terminal domain, resulting in its degradation through the ubiquitin-proteasome system. We also show that NCT-80 offers reasonable therapeutic efficacy, including significant suppression of viability, colony forming abilities, and sphere-forming capacities of a panel of NSCLC cell lines and their drug resistant sublines *in vitro* and the growth of NSCLC PDX tumors. Given that liver and ocular toxicities are major drawbacks of currently available Hsp90 inhibitors 66, the prominent safety profile of NCT-80 seems to be clinically favorable. Importantly, NCT-80 exhibited no detectable toxicity in several normal cells derived from different organs; NCT-80 treatment at therapeutic doses was well tolerated in mice without any detectable inflammation and injury in major organs, including the lung, liver, kidney, and brain.

Of note, we observed inhibition of PD-L1 expression in NSCLC cells resistant to anticancer therapies by treatment with NCT-80. PD-L1 plays an important role in the evasion of immune surveillance and activation of intrinsic prosurvival pathways 51. Moreover, some PD-L1-regulating transcription factors (e.g., HIF-1α and STAT3) are Hsp90 client proteins 46; several anticancer therapeutics are known to elevate PD-L1 level as a drug resistance mechanism 67, 68. Considering the aforementioned points, Hsp90 inhibition may potentiate antitumor effects of chemo- or molecular targeted therapies by downregulating PD-L1 expression. We also found that combinatorial treatment with NCT-80 increased the antitumor effect of chemotherapy (paclitaxel and cisplatin in combination) along with decrease in PD-L1 expression and increase in caspase-3 cleavage *in vivo*. Although additional studies are required to investigate the underlying mechanism, this result suggests the potential utility of NCT-80 as an adjuvant therapy for enhancing the antitumor effect of chemotherapeutic agents. Meanwhile, in contrast to the considerable antitumor effect of NCT-80 in the PDX model, we observed relatively modest antitumor effect of NCT-80 as a monotherapy in the LLC-Luc allograft model. Reduced antitumor effect of NCT-80 in the LLC-Luc syngeneic mouse model might be due to the highly tumorigenic and metastatic nature of LLC cells 69. In addition, the NOD/SCID mice, an immunocompromised mouse strain used in the PDX model, are defective in B and T lymphocytes and possess less functionally mature macrophages 70. Therefore, the blunted antitumor effect of NCT-80 in the syngeneic mouse model might also be caused by immune-suppressive tumor environments, created by regulatory lymphocytes (Treg and Breg), myeloid-derived suppressor cells, and tumor-associated macrophages. PD-1, a PD-L1 receptor, has been known to be expressed at the surface of a number of immune cells including activated T cells, natural killer (NK) cells, B cells, macrophages, dendritic cells, and lymphocytes (T cells and B cells) with regulatory phenotypes (Tregs and Bregs) 71-73; therefore, the tumor microenvironment consisting of these PD-1-expressing immune cells in the syngeneic mouse model may be beyond the capacity of NCT-80 to enhance antitumor immunity by suppressing PD-L1 expression.

## Conclusions

Here, we show that treatment with Hsp90 inhibitor activates IL-6/STAT3 signaling through the actions of Akt and ERK followed by transcriptional upregulation of Wnt ligands. The subsequent activation of Wnt signaling pathway enables NSCLC cells to acquire CSC phenotypes and survival capacities against Hsp90 inhibitors. Our findings further provide evidence that NCT-80 binds to the C-terminal domain of Hsp90, disrupts Hsp90-STAT3 interaction, and induces STAT3 degradation without evoking the Wnt-mediated CSC-like properties. NCT-80 effectively inhibits both CSC and non-CSC populations of NSCLC, eventually leading to potent antitumor activities with minimal *in vitro* or *in vivo* toxicity. These results suggest that NCT-80 can be considered a novel hit compound for further development as an anticancer Hsp90 inhibitor. Further studies are warranted to investigate the effectiveness of NCT-80 in additional preclinical and clinical settings.

## Supplementary Material

Supplementary figure and table.

## Figures and Tables

**Table 1:**
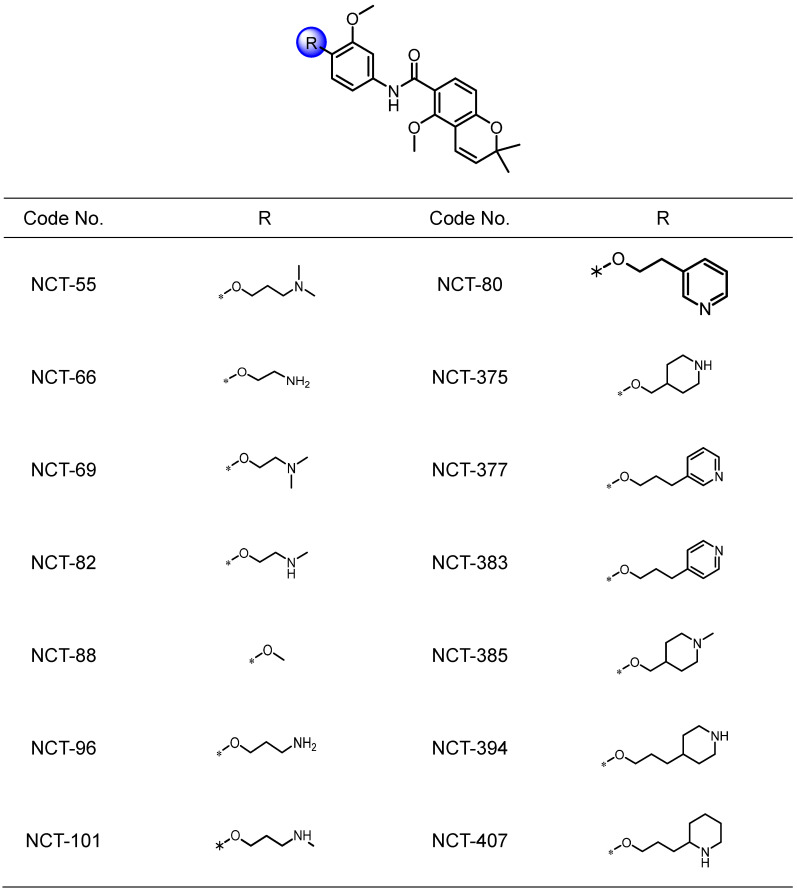
Structures of NCT series compounds.

## Abbreviations

17-AAG: 17-allylamino-17-demethoxygeldanamycin
AD: anchorage-dependent
AID: anchorage-independent
BUN: blood urea nitrogen
Crea: creatinine
EGFR: epidermal growth factor receptor
CE: cytoplasmic extracts
Con: control
Cl-Cas3: cleaved caspase-3
Cl-PARP: cleaved poly-(ADP-ribose) polymerase
CM: conditioned medium
CSC: cancer stem cells
CsR: cisplatin-resistant cells
CHX: cycloheximide
DARTS: drug affinity responsive target stability
ELDA: extreme limiting dilution analysis
EMT: epithelial-mesenchymal transition
ER: erlotinib-resistant cells
EV: empty vector
GOT: glutamate oxaloacetate transaminase
GPT: glutamate pyruvate transaminase
Hsp90: heat shock protein 90
LLC: Lewis lung carcinoma
LRP6: LDL Receptor Related Protein 6
NE: nuclear extracts
NSCLC: non-small cell lung cancer
NOD/SCID: non-obese diabetic-severe combined immunodeficiency
PARP: poly-(ADP-ribose) polymerase
PCNA: proliferating cell nuclear antigen
PD-1: programmed cell death protein-1
PD-L1: programmed cell death ligand 1
PDX: patient-derived tumor xenograft
PcR: paclitaxel-resistant cells
PmR: pemetrexed-resistant cells
RBC: red blood cells
RLU: relative luciferase unit
ROP: retro-orbital puncture
SD: standard deviation
STAT3: signal transducer and activator of transcription 3
TKI: tyrosine kinase inhibitor
WB: Western blot
WBC: white blood cells
