# Supplementary material for: A novel C-terminal heat shock protein 90 inhibitor that overcomes STAT3-Wnt-β-catenin signaling-mediated drug resistance and adverse effects
Source: Theranostics. 2022 Jan 1;12(1):105–25. doi: 10.7150/thno.63788 (PMC8690924; doi:10.7150/thno.63788)
Supplement: Supplementary file 1 — Supplementary figure and table. [file thnov12p0105s1.pdf]

Table S1. Primer sequences used in this study.

Gene	Forward Sequence (5'-3')	Reverse Sequence (5'-3')
WNT1	TGATACGCCAAAATCCGGGG	AGCCTCGGTTGACGATCTTG
WNT2	ACTCTCAGGACATGCTGGCT	ACGAGGTCATTTTTCGTTGG
WNT3	TGTGAGGTGAAGACCTGCTG	AAAGTTGGGGGAGTTCTCGT
LRP6	CCCATGCCCCTGGTTCTACT	CCAAGCCACAGGGATACAGT
MYC	GAGCCTGCCTCTTTTCCACA	CAGCGACTCTGAGGAGGAAC
BCL2	CTGCACCTGACGCCCTTCACC	CACATGACCCCACCGAACTCAAAGA
CCND1	AAGTGCGTGCAGAAGGAGAT	TTAGAGGCCACGAACATGC
BIRC5	AGAACTGGCCCTTCTTGGAGG	CTTTTTATGTTCCTCTATGGGGTC
AXIN2	ACTGCCCACACGATAAGGAG	CTGGCTATGTCTTTGGACCA
DNMT1	CCATCAGGCATTCTACCA	CGTTCTCCTTGTCTTCTCT
CD44	CAATAGCACCTTGCCCACAAT	AATCACCACGTGCCCTTCTATGG
YAP1	GCAACTCCAACCAGCAGCAA	CGCAGCCTCTCCTTCTCCAT
SNAI2	TGTTGCAGTGAGGGCAAGAA	GACCCTGGTTGCTTCAAGGA
TWIST1	GGACAAGCTGAGCAAGAT	CTCTGGAGGACCTGGTAG
VIM	CGGCTGCGAGAGAAATTGC	CCACTTTCCGTTCAAGGTCAAG
CDH2	GGTGGAGGAGAAGAAGACCAG	GGCATCAGGCTCCACAGT
NANOG	CCTCCTCCATGGATCTGCTTATTCA	CAGGTCTTCACCTGTTTGTAG
POU5F1	TGCAGCAGATCAGCCACATC	CTCGGACCACATCCTTCTCG
SOX2	AACCAGCGCATGGACAGTTA	ATCATGCTGTAGCTGCCGTT
ACTB	TCATTCCAAATATGAGATGCGTTG	TAGAGAGAAGTGGGGTGGCT
RN18S	CGCCGCTAGAGGTGAAATTC	TTGGCAAATGCTTTCGCTC

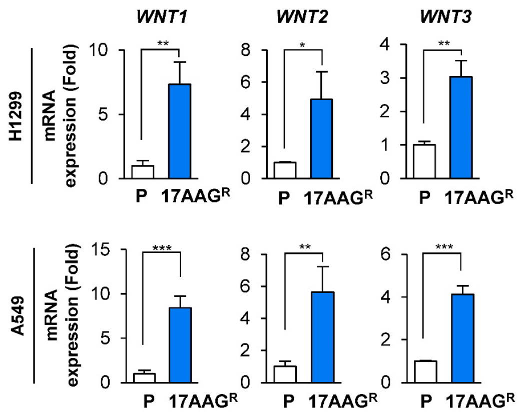

Figure S1. Regulation of mRNA expression of Wnt ligands in 17-AAG-resistant cells. Real-time PCR analysis showing changes in mRNA expression of Wnt ligands in 17-AAG-resistant (17AAG^R) H1299 and A549 cells by comparison with those in the corresponding parental (P) cells. The bars represent mean ± SD; *P < 0.05, **P < 0.01, and ***P < 0.001, as determined by a two-tailed Student's t-test in comparison with the indicated control.
